# Examining burnout in the electrical sector in Ontario, Canada: A cross-sectional study

**DOI:** 10.3934/publichealth.2023060

**Published:** 2023-11-14

**Authors:** Behdin Nowrouzi-Kia, Ali Bani-Fatemi, Aaron Howe, Simrat Ubhi, Mitchel Morrison, Harseerat Saini, Vijay Kumar Chattu

**Affiliations:** 1 Restore Lab, Department of Occupational Science and Occupational Therapy, Temerty Faculty of Medicine, University of Toronto, Ontario, Canada; 2 Centre for Research in Occupational Safety and Health, School of Rural and Northern Health, Laurentian University, Sudbury, Ontario, Canada; 3 Rehabilitation Sciences Institute, Temerty Faculty of Medicine, University of Toronto, Toronto, Ontario, Canada; 4 Department of Psychology, University of Toronto, Scarborough, Ontario, Canada; 5 Department of Physics and Astronomy, University of Waterloo, Waterloo, Ontario, Canada; 6 Center for Global Health Research, Saveetha Medical College and Hospitals, Saveetha Institute of Medical and Technical Sciences, Saveetha University, Chennai, India; 7 Center for Evidence-based Strategies, Global Health Research and Innovations Canada Inc (GHRIC), ON, Toronto

**Keywords:** electricians, skilled trades, workplace, mental health, Canada

## Abstract

Workers in the trades sectors often experience mental health issues and decreased work ability due to occupational stress, workplace hazards and living in danger or constant fear of injury. Understanding the impacts of psychosocial risk factors on construction workers' mental health can aid in decreasing workplace injuries, lessening disabilities and increasing worker productivity. In this study, we focus on understanding and assessing the mental health and wellness of individuals in the electrical sector that are members of the Employer Engagement Project (EEP) from the Ontario Electrical League (OEL). The subset of potential participants included electricians and plumbers in Ontario working for small to medium sized employers (SME). The recruitment took place in 2022, with a total of 82 participants who completed a survey collecting demographic information, assessing the importance and availability/satisfaction of workplace factors and stress-and burnout-related questions. The data were analyzed using IBM SPSS Statistics 29.0. Two-sample Mann-Whitney U tests were performed to test for associations between the availability of work-related factors and burnout scores among the participants. Burnout scores were determined using the Copenhagen Burnout Inventory. Our findings demonstrate that dissatisfaction of the following factors: Workload allocation, internal staff development opportunity and stable staffing/minimal turnover, were associated with high burnout levels. The findings indicate there may be a relationship between certain work-related factors and burnout levels experienced. There is a need for improvement of workload allocation in SMEs to help enhance the mental health and well-being of employees.

## 1. Introduction

### 1.1. Background

Employment can provide individuals with financial assurance [1], accessibility to resources and support measures [2] and the essential necessities required to sustain themselves and their families. However, working can also lead to life-changing illnesses, which can often outweigh the harm associated with work-related injuries. As reported by the Association of Workers' Compensation Boards of Canada (2021) 13553 trades workers died as a result of work-related diseases, with 1103 workers in this group dying as a result of mental disorders or syndromes [3]. Additionally, the Construction Industry Rehabilitation Plan of British Columbia, Canada revealed that 83% of construction workers in the study experienced moderate to severe levels of mental health issues, putting them at a higher risk of developing mental illnesses [4].

Construction workers often face mental health issues, including increases in anxiety, depression and suicidal ideation, often resulting in suicide [5],[6]. Jacobsen and colleagues (2013) collected data on the mental wellness of construction workers using rapid mental health surveys [7],[8]. Clinical interviews were subsequently performed on a subset of participants (*N* = 10) as a follow-up to the mental health survey, which revealed that 90% of the construction workers interviewed met the criteria for a mental disorder diagnosis. Furthermore, research from Stocks et al. (2010) indicated that construction workers had higher levels of post-traumatic stress due to the workplace hazards and the dangers associated with construction jobs [8].

Researchers who have examined the mental health of electrical workers have found that dangerous living conditions have substantial impacts on worker's mental health [9]–[19]. The constant fear and exposure to dangers play a significant role in workers' mental well-being [15]. These dangers include lethal electrical shock, electrical smoulders and exposure to fire, lead and explosions. Additionally, the physical impacts of electrical mishaps and work-related injuries can have lasting effects on one's workability related to long-term cognitive and emotional damages [16]. Sauter et al. (1998) investigated the effects that privatization, transitioning from public to private domains, on the mental health of electrical workers. We established that work-related stress and tension were often associated with insomnia and nervousness in workers, demonstrating that workplace organization and performance often impacts on workers' illnesses [13]. Outcomes such as work-related stress and workloads can also be impacted by the current labor shortages in the skilled trades industry. As reported by the Canadian Apprenticeship Forum, approximately 75,000 new apprentices need to be hired between 2022–2027 to meet the gap of skilled workers in Red Seal trades [20]. Furthermore, the Government of Canada reported that 2020 had the largest declines of new apprentice registrations and certifications since 1991. Showing a decrease of 28.5% in new apprenticeship registrations and a decrease of 31.5% in receiving trade certifications since 2019 [20].

Trades workers often struggle to cope with suicidal ideation as a result of mental health issues [5],[6]. Male construction workers represent the occupational group that is at the most risk of committing suicide [21]. Current research indicates that the rate of suicide among younger, inexperienced male construction workers is double that of the average younger male, partially due to the segregation and bullying between males within the trades industry [22]. Additionally, the number of reported suicides between 2011 and 2015 were 3.7 times higher than the UK national average, with a total of 1419 reported suicide cases [5]. The second largest cause of death for workers within the construction industry of British Columbia, Canada was reported to be suicide with a majority of instances involving men between 40–59 years of age [23]. Similarly, the rate of suicide by construction workers in Australia is double that of the national average of Australia [24],[25]. Regrettably, Canadian research and literature surrounding this subject lack depth, understanding, relevant data and are limited. This study will therefore adopt an exploratory design and aim to contribute to the limited Canadian literature while focusing on the mental health of electricians and its work-related factors.

Research shows that psychological stressors, such as “mobbing” in the workplace, accompanied by associated factors such as substance abuse, interpersonal issues, financial problems, job stability, gender norms, cultural barriers (e.g., suppressing emotions/hyper-independence) and injury rates can increase the possibility of developing mental illnesses [25]. Roughly 400000 workdays are effectively missed each year in the United Kingdom due to mental health issues within the construction industry [5]. Monitoring and understanding the influence of psychosocial risk factors on workers' mental health can significantly decrease occupational injuries, limit disabilities and boost workers' productivity [25]. Mental health issues within the skilled trades, namely electrical and plumbing fields, have large economic consequences such as missed time from work and poverty, which has a bidirectional effect with mental health (i.e., poverty can be a cause and or a consequence of mental health issues) [26],[20].

The understanding of mental health issues among skilled trades workers is especially important considering that occupational stressors have significant impacts on the workability of electricians [27]. Work Ability Index (WAI) is a measurement tool that quantifies the duration of time one is able to work using evidence-based information. Work ability, as defined by the WAI, is the balance between human resources and work demands [27]. Kulić and associates (2019) utilized the Work Ability Index questionnaire to demonstrate that 11.5% of electricians exhibit poor workability, 25.0% moderate workability, 26.9% good workability and 36.5% excellent workability [28]. Burnout is described as a feeling of psychological and bodily weariness caused by prolonged instances of stress, most commonly in job-related demanding conditions or excessive obligations in one's daily life [29]. Using the Copenhagen Burnout Inventory, the authors found that electricians displayed higher scores than control groups and significantly higher workability index scores. The authors concluded that the strongest and most prevalent stressors that electricians encounter in their workplace consists of factors including work scheduling (e.g., night shifts, working overtime), job performance demands (e.g., meeting deadlines/time limits, heavy workloads), safety concerns (e.g., fear of injury, hazardous work environment) and unforeseen circumstances [29].

### 1.2. Related works

The prevalence of mental health issues in heavy labor industries, such as electrical and plumbing, have large consequences; however, the research in this area is dated and lacking in the electrical sector. Apprentices within Australia continue to experience extensive pressure to meet unrealistic expectations within the industry. Apprentices often report experiencing workplace bullying, a feeling of being positioned at the “bottom of the food chain”, verbal abuse and exposure to derogatory language in the workplace. With such poor work environments, many newcomers have the expectation of requiring resilience and “thick skin” to endure such conditions [30].

Countries such as Australia and the United Kingdom, as discussed, have put forward more diligent efforts to examine individuals' mental health in the electrical sector. They have examined factors such as suicide rates, mental health factors and workplace bullying, and reflect the experiences of apprentices and retiring workers in the trades industry. However, the Canadian literature surrounding these subjects lacks depth, understanding, relevant data and is unfortunately limited due to lack of research on this topic. With the statistics and relevant literature that has been developed in countries like Australia and the United Kingdom however, it is necessary to recognize there is a correlation between workplace mental health and worker burnout. This correlation has been linked to increases in suicide rates and experiences with unhealthy mentalities of workers in the industry. This further reveals the need for investigating the relationship between burnout and mental health outcomes in skilled trades workers in Canada to implement policies promoting worker mental health and strengthening overall worker wellness. We will, therefore, take on an exploratory design with a strong focus on the mental health of electricians and work-related factors aiming to contribute to the lacking Canadian literature.

### 1.3. Study's purpose

Our aim of this study was to investigate the mental health and well-being of trades workers under the OEL's EEP to implement workplace mental health policies, measures and resources. More specifically, the objective of this study was to explore an association between burnout, as a measure of mental health, and the availability of various work-related factors by reporting corresponding evidence.

## 2. Materials and methods

### 2.1. Setting

The OEL is a not-for-profit organization which represents the electrical industry of Ontario, Canada [31]. With over 38000 province-wide partners, OEL aims to foster, bolster and advocate current industrial issues. Issues within the industry are presented to government officials via conferences, training programs and government liaison programs.

The OEL aims to accomplish a 1:1 apprenticeship to mentor ratio to support apprentices and employers in apprenticeship training using their mentorship model, which was developed in their 2018–2019 EEP [31]. Employers often require extra support to ensure training opportunities and a sustainable workforce for Ontario's trades workers. This is often due to the fact that electrical apprenticeships are a lengthy process, requiring five or more years to complete. The recent efforts made towards a 1:1 ratio have focused on removing the restrictive requirements surrounding the number of licensed electricians and/or plumbers needed per every new apprentice [31]. The 1:1 apprenticeship ratio has allowed for new opportunities and provided support for employers that were previously unable to hire and train apprentices due to the ratio's restrictions.

The EEP project works towards offering a solution to the shortage in skilled trades by providing electrical and plumbing employers with the means to train their apprentices and bridge the skills gap within the industry. The OEL and the University of Toronto (UofT) have partnered to evaluate the EEP project using qualitative and quantitative analyses to assess the strength of existing supports and identify potential future supports for apprentices and employers. Additionally, mental health and well-being evaluations were performed on the employers of electrical and plumbing companies engaged in the EEP project. This study protocol was approved by the University of Toronto's Institutional Research Ethics Board (protocol number: 41519). Informed consent was collected from all subjects participating in the study. All participants have given their written informed consent for this work to be published.

### 2.2. Study design

A mixed-methods, sequential design was employed in this study to allow for better understanding of the employer mentorship program and EEP project provided by OEL, as well as their outcomes in the trades industry. We utilized quantitative methods and an evidence-based approach to measure and assess the OEL's mentorship program's effectiveness using data collected from SMEs. We virtually conducted two focus group discussions with 11 SMEs, each lasting approximately 60–70 minutes.

### 2.3. Participants

We investigated the mental health outcomes of a subset of Ontario's electricians and plumbers working for small-to-medium contractors, which consist of less than 50 employees. The OEL used external service providers to further develop its pilot project methods and incorporated pre-screening technologies as a means of initial contact with the participants. Following this, OEL staff took part in conducting in-depth interviews which examined employer's willingness to participate in the project to determine its target employers. The University of Toronto proceeded to assess the referred employers for their mental well-being and workplace stressors. The participants of the study (*n* = 82) were recruited in 2022 consisting of OEL members participating in the EEP project. Inclusion criteria are as follows: SMEs (i.e., less than 50 employees), target age range of 18 to 70 years, enrolled in the EEP project, proficient in English and the ability to provide research consent. The OEL provided resources in the mentorship program which consisted of mentorship training, tools for hiring and employer outreach. Employer outreach was accomplished by reaching out to employers that were not presently involved in apprenticeship training and offering them information and resources about the advantages of apprentice training and useful business tactics.

### 2.4. Data collection and survey

The participants of the study were recruited by the OEL to complete an online survey using an email script prepared by the University of Toronto. All participants provided informed consent to participate in the study. Trained research staff administered the validated questionnaires, that were based on work done by the primary investigator (B.N.K.) [32]. The survey measure contained 38-item questions that allowed for the collection of demographic data from the participants. The survey was distributed to participants online over a 12-week period with the survey taking approximately 25 minutes to complete. Demographic data collected included; participant's gender, sex, age, ethnic background, marital status, completion of education, total number of years working, number of years working as an electrician/plumber, total hours worked on a daily and weekly basis, hours worked as overtime, total income and the commute/travel time. Additionally, the questionnaire incorporated occupational stress and burnout factors characterized by the Copenhagen Burnout Inventory (CBI) [33] as well as the National Institute for Occupational Safety and Health (NIOSH) and the Generic Job Stress Questionnaire [34].

The CBI is a valid and reliable tool used to measure and determine the degree of burnout within various work sectors. It is a normed measure that is often used in both clinical and research settings due to its simple and accessible nature [35]. The CBI can be broken down into three subscales known as: personal burnout, work-related burnout and colleague-related burnout. Personal burnout can be described as the level of bodily and mental exhaustion a worker experiences. Similarly, work-related burnout is the level of bodily and mental exhaustion one endures that is related to or caused by work. Finally, colleague-related burnout refers to the level of bodily and mental weariness one feels while working with colleagues. Throughout many studies, the measure shows reliable and valid psychometric properties that accurately measure and assess occupational burnout [36]–[40]. A higher score was indicative of high degree of burnout, whilst a lower score suggested lower burnout levels and greater well-being. Furthermore, the personal burnout measure enabled an extensive examination of burnout levels across all participants, irrespective of job position.

The NIOSH Generic Job Stress Questionnaire is a measure of the major factors influencing occupational stress. These factors include: Physical environment, conflicts with the role, issues with control, co-worker support, administrative support, workload and work demands [34]. The NIOSH questionnaire is used internationally for data collection and analyses pertaining to occupational stress. Additionally, it focuses on stressors leading to occupational strain and factors affecting how workers cope with stressors.

### 2.5. Data analysis

The quantitative data provided descriptive statistics through calculation of means, standard deviations, percentages and grouped totals through Excel. The importance of and availability/satisfaction of various factors in the workplace were demonstrated through counts and percentages for each factor. Analysis of association was performed using IBM SPSS Statistics 29.0 for Windows [41]. The examination of a potential relationship between the availability/satisfaction of workplace factors (i.e., career advancement possibility) and burnout scores of different types, obtained from the CBI questionnaire results, was performed using non-parametric Mann-Whitney U tests. This was used to test the relationship between two independent samples, therefore allowing us to understand the association between multiple survey categories.

For the availability of each work-related factor, a participant responds with one of three potential responses: (i) Not available, (ii) available but needs improvement and (iii) available to my satisfaction. The percentage of individuals reporting their satisfaction with the factor was interpreted as the percentage of participants also satisfied with the availability of the factors. Moreover, “effect size” was introduced as the difference in average burnout score of a certain type (e.g., personal burnout) between the individuals who were not satisfied with and those who were satisfied with the availability of the given work-related factor. A positive effect size would indicate that the average burnout score of those who are dissatisfied with the work-related factor is greater than the average burnout score individuals who were satisfied with the factor. The association between the effect size of each work-related factor and the percentage of individuals satisfied with each factor were investigated to understand a possible correlation, which would explain the different types of burnout.

The use of non-parametric Mann-Whitney U tests helped to determine the level of correlation between the two calculated results. The samples were tested to an alpha level of 0.05 to understand the differing levels of significance between each burnout association. The percent satisfaction was paired with the association of burnout scores to identify factors associated with higher burnout and lower performance, with the idea that these factors can be improved to lessen the burnout amongst individuals working in these sector(s).

## 3. Results

### 3.1. Demographic characteristics of the study participants

The socio-demographic characteristics of the study's participants is summarized in Table 1. Despite some individuals having left some questions unanswered, all participants completed the demographic survey. To compensate for non-responses, the percentage of responses for a certain categorical value is considered out of the total number of responses to that question. There were 82 participants with 76 of them (92.7%) being male. The average age was 41.6 ± 15.1 years, with a range of 17 to 77. Of the 82 participants, it was found that 89.0% (*n* = 73) were born and/or raised in Ontario, while 97.6% (*n* = 80) obtained their training in Ontario. Over half of the participants (58.5%) described their highest level of education obtained as a college certificate/diploma, with 26.8% completing high school, 6.1% obtaining a university degree of any sort, and 8.5% listed as other, which includes sources of education such as apprenticeship and/or license (e.g., electrician). Most participants were primarily employed in the electrical sector (*n* = 77), with three individuals employed in the plumbing sector and 2 individuals employed in both sectors. Of the 82 participants, only 19.5% (*n* = 16) belong to a union. As for the income, 14 did not report their gross annual income, while 50% (*n* = 34) of the responding participants indicated their gross annual income is less than $80,000 and 50% (*n* = 34) reported greater than $80,000. Conclusively, one participant recently retired (i.e., in 2019) and five participants were planning to retire within the next five years. In total, 11 participants (i.e., 14.7%) indicated that they do not plan to remain in their present role for the next 5 years.

**Table 1.** Demographic characteristics of study participants (*n* = 82).

**Demographic variables**		**Frequency (*n*)**	**Percent (%)**
**Age (years)**	Mean	41.6	
	Standard deviation	15.1	
	Range	17–77	
**Gender**	Male	76	92.7
	Female	6	7.3
**Marital status**	Married/Common-law	58	70.7
	Single	21	25.6
	Other*	3	3.7
**Education level**	University undergraduate and graduate	5	6.1
	College certificate/diploma	48	58.5
	Completed high school	22	26.8
	Other	7	8.5
**Primary language**	English	77	93.9
	French	1	1.2
**Ethnicity**	White	67	83.8
	Asian	3	3.8
	Other***	10	12.5
**Smoking**	Non-smoker	55	67.1
	Smoker/Former Smoker	27	32.9
**Current employment**	Employed in electrical sector	77	93.9
	Employed in plumbing sector	3	3.7
	Employed in both electrical and plumbing sectors	2	2.4
**Belong to a Union**	Yes	16	19.5
	No	66	80.5
**Average overtime hours per week**	None	37	51.4
	5 hours or less	19	26.4
	6–10 hours	10	13.9
	More than 10 hours	6	8.3

Notes: *Includes divorced/separated and widowed. **Includes Ukrainian, Romanian and Arabic. ***Includes Indian Caribbean, mixed (white and aboriginal) and mixed (other).

### 3.2. Importance of work-related factors

**Figure 1.**
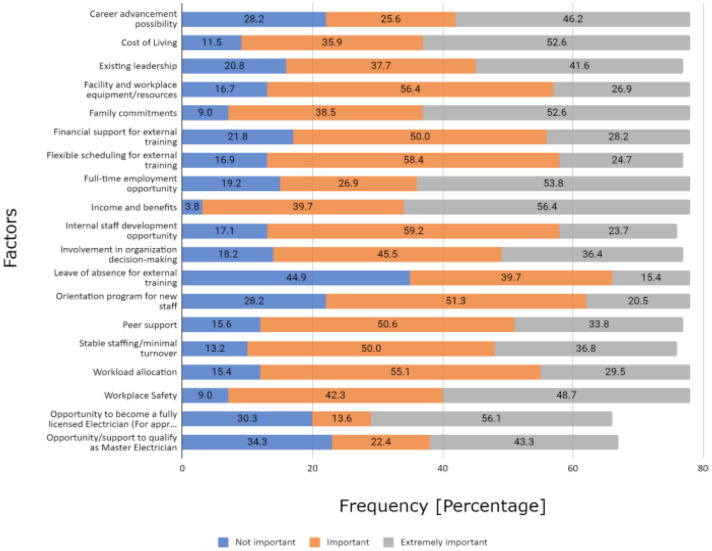
Importance of the work-related factors keeping participants working in their present position at their current place of work.

Among the surveyed participants, income and benefits (56.4%), opportunity to become a fully licensed electrician (56.1%), full-time employment opportunity (53.8%), family commitments (52.6%) and cost of living (52.6%) were the most essential factors keeping participants in their current position. This was indicated by the higher frequency of individuals who labelled these factors as extremely important. Contrarily, leave of absence for external training (44.9%), opportunity/support to qualify as master electrician (34.3%) and an opportunity to become a fully licensed electrician (30.3%) were among the factors of least importance to the participants (Figure 1). Note that the opportunity to become a fully licensed electrician falls on both ends of the importance scale due to high density of responses in the “Not Important” and “Extremely Important” categories, with only 13.6% of responses indicating “Important.”

### 3.3. Availability and satisfaction of work-related factors

**Figure 2.**
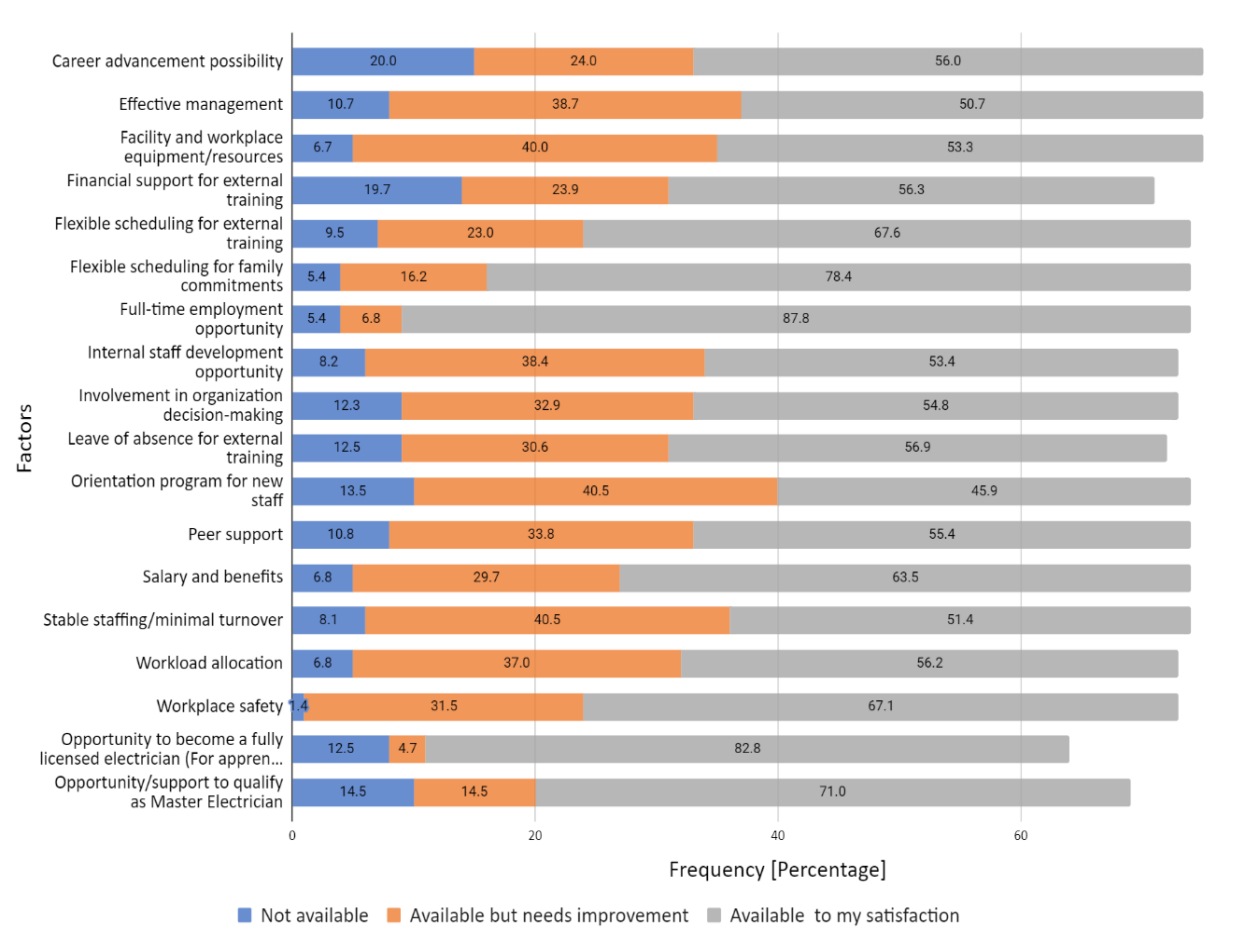
Availability and satisfaction with work-related factors.

Full-time employment opportunity (87.8%), opportunity to become a fully licensed electrician (82.8%) and flexible scheduling for family commitments (78.4%) were found to be the work-related factors which yielded the highest proportion of participants thus indicating that these factors are available to their satisfaction. While 20.0% of individuals reported that career advancement possibility is not available, and 19.7% reported that financial support for external training is not available (Figure 2). Moreover, orientation program for new staff yielded the highest percent unsatisfied (54.1%), considering the totals for “Not Available” and “Available but Needs Improvement” combined as a total of individuals unsatisfied.

### 3.4. Burnout

In each category of burnout, (i.e., personal, work-related, colleague-related and total burnout), the mean score of each participants' results were gathered. An average score of 50–74 is regarded as a moderate level of burnout, 75–99 is deemed high burnout, whilst an average score of 100 is considered severe burnout [33]. The total mean score was 32.34 ± 16.84. Of the 82 participants, there were 22 cases of moderate personal burnout, 15 with moderate work-related burnout and 10 individuals with moderate colleague-related burnout. Moreover, there were five individuals with high personal burnout and one with high colleague-related burnout. Conclusively, there was one case of severe burnout, in the work-related category. Table 2 contains a summary of the CBI analysis.

### 3.5. Association between work-related factors and burnout

**Figure 3.**
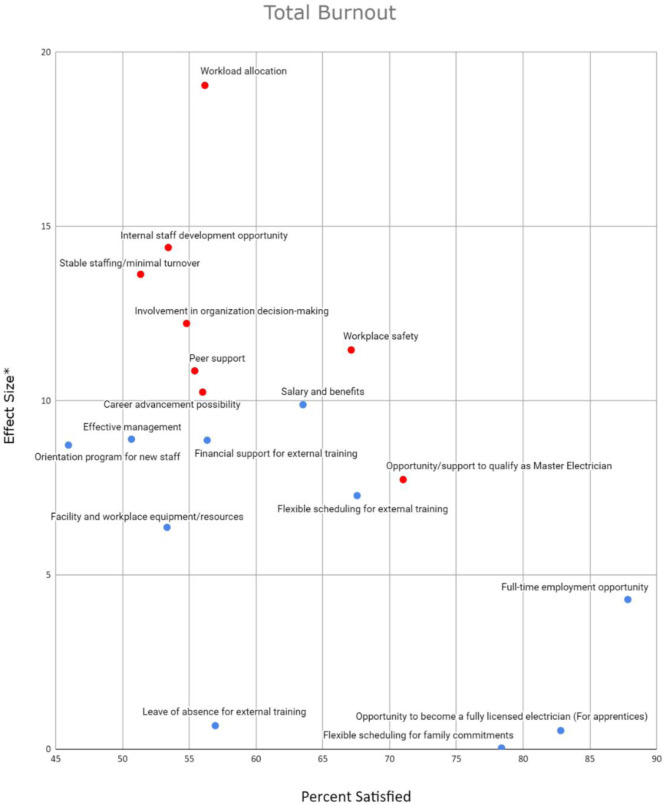
Impact of availability of work-related factors on total burnout. *Effect size for a given work-related factor is calculated as the difference in average score of the individuals who are not satisfied with the factor and those who are satisfied with the factor. Red dots indicate the relationship between satisfaction with the factor and their average burnout score is significant to a 0.05 alpha level. Blue dots indicate the relationship is not significant to a 0.05 alpha level.

Figure 3 demonstrates the association between availability/satisfaction between different work-related factors and total burnout, as described by the CBI scale. The figures highlight “effect size” vs the percentage of individuals who were satisfied with the work-related factor. The effect size is the difference in the average burnout score of those satisfied with the factor and those who are not satisfied, who have indicated the factor could be improved or that it is not available. All questions from the CBI were included in the average score, combining all three types of burnout. Thus, a larger effect size indicates higher burnout in those dissatisfied with the factor. A higher percentage satisfied indicates that a larger proportion of individuals selected that the factor is available to their satisfaction. Lastly, associations that were not deemed significant to a 0.05 alpha level are plotted in blue, and associations deemed significant to a 0.05 alpha level are plotted in red.

**Table 2.** Results of the Copenhagen Burnout Inventory Questionnaire (*N* = 82).

**Type of Burnout**	**Personal**	**Work-related**	**Colleague-related**	**Total**
**Mean [*SD*]**	40.51 [19.95]	33.02 [19.04]	23.29 [19.40]	32.34 [16.84]
**Minimum score**	4.17	3.57	0.00	6.58
**Maximum score**	87.50	100.00	75.00	88.16
**Median**	37.50	28.57	25.00	28.95
**Moderate burnout (*n*)**	22	15	10	11
**High burnout (*n*)**	5	0	1	1
**Severe burnout (*n*)**	0	1	0	0

Figure 3 suggests that workload allocation is a work-related factor that could be improved to allow for greater satisfaction rates in order to decrease burnout of all types. Each of the four plots indicate that dissatisfaction with workload allocation is associated with much higher burnout, on average. With a relatively low satisfaction rate of 56.2%, greater focus on this factor may result in a reduction of burnout.

Similarly, based on the plot, stable staffing/minimal turnover and internal staff development opportunity are two other factors with relatively low satisfaction rates (both less than 60%) and a large effect size under significant association. This could indicate that focus should be put on these factors to attempt at improving the availability of the factor in the workplace in order to decrease burnout in individuals.

Furthermore, Figure S1, Figure S2 and Figure S3, which can be found in the supplementary materials, showcase the association between the satisfaction of work-related factors and the different types of burnout (e.g., personal). These plots are consistent with Figure 3, demonstrating similar tendencies.

## 4. Discussion

There were 82 participants surveyed in this study. The data was quantitatively analyzed to examine mental health outcomes among the individuals surveyed. We investigated demographic variables, the importance of certain work-related factors and the availability/satisfaction of workplace factors and how these relate to the levels of burnout experienced by the participants.

The study consisted of 76 males and 6 females, with 93.9% being employed primarily in this sector. Satisfaction rates were investigated alongside CBI burnout scores of the individuals to garner an understanding of a potential factor and burnout association.

There is great need for investigating stress and burnout in the skilled trades industry, alongside a need for improvement of recruitment and retention in the industry to combat the current labour shortages. Construction job vacancies in Canada increased by 46.7% to 62600 in the second quarter of 2021 [42]. The OEL employer mentorship program, which was created to assist employers in training apprentices and encouraging future tradesmanship, might be useful in preventing these numbers from increasing in the coming years since our previous qualitative findings demonstrated that the employers valued this program [43]. The survey data indicated several cases of moderate burnout, with many being labelled under personal burnout, as well as some cases of high burnout (personal, colleague-related), and even one case of severe burnout (work-related). The number of individuals who have experienced burnout to any degree can be related to varying factors in the workplace, such as the association found between higher burnout scores and dissatisfaction with the workload allocation factor. It also depends on the method in which burnout was assessed, which was conducted with CBI.

While some studies have been conducted to examine the impact of job-related parameters on the well-being of skilled tradesmen, the existing literature focusing on the mental health of electricians and plumbers is minimal. Thus, this study is important as it provides an understanding of the difficulties that employees in this field experience, underlining the importance of effective burnout interventions. It serves as a helpful resource for corporations and policymakers as it helps them to recognize and understand the specific psychological concerns of these workers. With this knowledge, they can implement essential reforms that prioritize employee well-being and foster supportive work environments, leading to a healthier and more productive workforce. This may include providing counselling services or resources for stress management.

This study has been conducted over a 2-year period, with data collection taking place from September 2022 to December 2022. Based on results from the first-year data, of the 40 participants, there were 7 individuals who experienced personal burnout to any degree, 2 who had experienced work-related burnout and 2 who had experienced colleague-related burnout [43]. In proportions, that is 17.5%, 5% and 5%, respectively. After the second year of survey results for each categorical burnout type, there has been a drastic increase in the percentage of participants who have experienced each type of burnout. For example, the percentage of individuals who experienced personal burnout has risen from 17.5% to 33.3% after completion of the second-year survey results. Moreover, for each burnout category, the mean CBI score has increased from year 1 to year 2, indicating a general increase in population burnout. See Table S2 in the supplementary materials for a full comparison of the data after one year to the data after two years of the study.

The rise in burnout among electricians and plumbers could be attributed to individual worries about the escalating cost of living caused by the COVID-19 crisis. Recent studies have found that families burdened with mortgage payments experienced lower perceived health and more mental anguish than those without mortgage obligations [44],[45]. Furthermore, the continuous influence of COVID-19 on the economy has resulted in the greatest inflation rates seen in the past years, resulting in higher food and transportation expenses. This circumstance, along with the prospect of an impending economic slump, might be the cause of the increase in the proportion of individuals suffering from personal burnout [46]. Additionally, there was one case of severe work-related burnout, which might be due to the demanding nature of that individual's skilled trades profession, which necessitates working in uncomfortable positions and performing physically exhausting tasks [47]–[50]. Individual traits such as response to stress (i.e., having poor coping and resilience abilities) and previously present mental health issues must also be considered. All these factors could have been additive to overburden the individual and predispose them to severe burnout.

In the present study, the majority of the individuals were male (92.7%), identified as white (83.8%) and were born and raised in Ontario (89%). These findings are consistent with the Canadian Job Bank data, which revealed that in the electrical and plumbing trades the presence of women is low [51],[52]. Furthermore, in 2016, immigrants made up 24% of the labor force in Canada [53], and the number of Aboriginal individuals among newly qualified journeypersons is comparable to their representation in the Canadian population [54]. However, Aboriginal (3.8%) and immigrant (11%) groups were underrepresented in this study's sample demographics, indicating a lack of diversity and minority individuals within the sample. Future research may use larger and more diversified samples that include different minority groups, while also conducting intersectional analysis through a gender-based approach.

As mentioned in the results section, based on the satisfaction and burnout association plots, lower satisfaction of some work-related factors was found to be associated with higher burnout. This would indicate that improving the availability of these work-related factors may decrease burnout among workers in the industry, improving the overall wellbeing of individuals. Two of the more prominent factors with a higher association of being linked to more burnout were stable staffing/minimal turnover and internal staff development opportunity. Hence, there should be a focus on improving this work-related factor to, in turn, improve the overall wellbeing of the individuals. Stable staffing and minimal turnover connect employee retention, providing an idea for a focus on retention in the industry. Some strategies to bolster retention might include fostering a positive work environment, and offering sufficient assistance and resources to employees. Conducting employee feedback surveys on a regular basis to uncover issues would show dedication to employee well-being. Additionally, offering staff development programs, such as regular training, skill enhancement initiatives and career advancement opportunities could improve job satisfaction, and overall wellness.

It should be noted that the study had limitations. First, the use of self-reported data to assess burnout. Each participant differs in their understanding of the questions and willingness to report truthfully. Furthermore, it also introduces subjectivity and possible bias as one might under-or over-report their burnout levels. Second, a relatively small sample size of 82 participants presented limitations with respect to the validity of generalized results for the industry. Third, the surveys were conducted over a 2-year span in which some individuals have recorded responses twice, one in each year, while the rest have completed the survey once. To address this problem, duplicate responses were removed and only their most recent responses were analyzed. However, similar to the subjectivity linked with self-reporting, this might also generate response bias. Additionally, due to a lack of psychometric research to evaluate the CBI's applicability in electricians and plumbers, there is a lack of validity and reliability of the CBI in measuring burnout among these groups.

## 5. Conclusions

The findings of this study indicate that there may be a relationship between the workers' satisfaction of work-related factors and the burnout level experienced by individuals in electrical and plumbing sectors of the Ontario construction industry. There is a need to focus on improvement and allocation of mental health resources in small-medium-sized employers to effectively address the shortages of apprentices and journey persons within the industry.

## Contributions

B.N.K. conceptualized the study and wrote the initial draft. M.M. completed data analysis as well as contributed to results and part of the discussion section. S.U. wrote the introduction, methods and parts of the discussion. H.S. completed the discussion section alongside Mitchel and Simrat. V.C., A.H., B.N.K. and A.B-F edited and reviewed the manuscript.

## Use of AI tools declaration

The authors declare they have not used artificial intelligence (AI) tools in the creation of this article.

## Supplementary

Figure S1, Figure S2, and Figure S3 examine the impact of availability of work-related factors on personal burnout, work-related burnout and colleague-related burnout, respectively. Table S1 discusses the association between the availability/satisfaction of work-related factors and the experience of burnout. Table S2 compares the burnout scores over a two-year period. In Table S2, the results after year 1 were obtained from Bani-Fatemi et al. (2022) under the same methodology, the results after year 2 were gathered from this study [43]. The consent form has been provided in the supplementary materials.
