# Supplementary material for: Examining burnout in the electrical sector in Ontario, Canada: A cross-sectional study
Source: AIMS Public Health. 2023 Nov 14;10(4):934–51. doi: 10.3934/publichealth.2023060 (PMC10764972; doi:10.3934/publichealth.2023060)
Supplement: Supplementary file 1 [file publichealth-10-04-060-s001.pdf]

AIMS Public Health

AIMS Public Health, 10(4): 934–951.
DOI: 10.3934/publichealth.2023060
Received: 21 July 2023
Revised: 27 October 2023
Accepted: 06 November 2023
Published: 14 November 2023

http://www.aimspress.com/journal/aimsph

Brief report

Examining burnout in the electrical sector in Ontario, Canada: A cross-sectional study

Behdin Nowrouzi-Kia[1,2,3,*], Ali Bani-Fatemi[1], Aaron Howe[1], Simrat Ubhi[1,4], Mitchel Morrison[1,5], Harseerat Saini[1] and Vijay Kumar Chattu[1,6,7]

[1] Restore Lab, Department of Occupational Science and Occupational Therapy, Temerty Faculty of Medicine, University of Toronto, Ontario, Canada
[2] Centre for Research in Occupational Safety and Health, School of Rural and Northern Health, Laurentian University, Sudbury, Ontario, Canada
[3] Rehabilitation Sciences Institute, Temerty Faculty of Medicine, University of Toronto, Toronto, Ontario, Canada
[4] Department of Psychology, University of Toronto, Scarborough, Ontario, Canada
[5] Department of Physics and Astronomy, University of Waterloo, Waterloo, Ontario, Canada
[6] Center for Global Health Research, Saveetha Medical College and Hospitals, Saveetha Institute of Medical and Technical Sciences, Saveetha University, Chennai, India
[7] Center for Evidence-based Strategies, Global Health Research and Innovations Canada Inc (GHRIC), ON, Toronto

* **Correspondence:** Email: behdin.nowrouzi.kia@utoronto.ca.

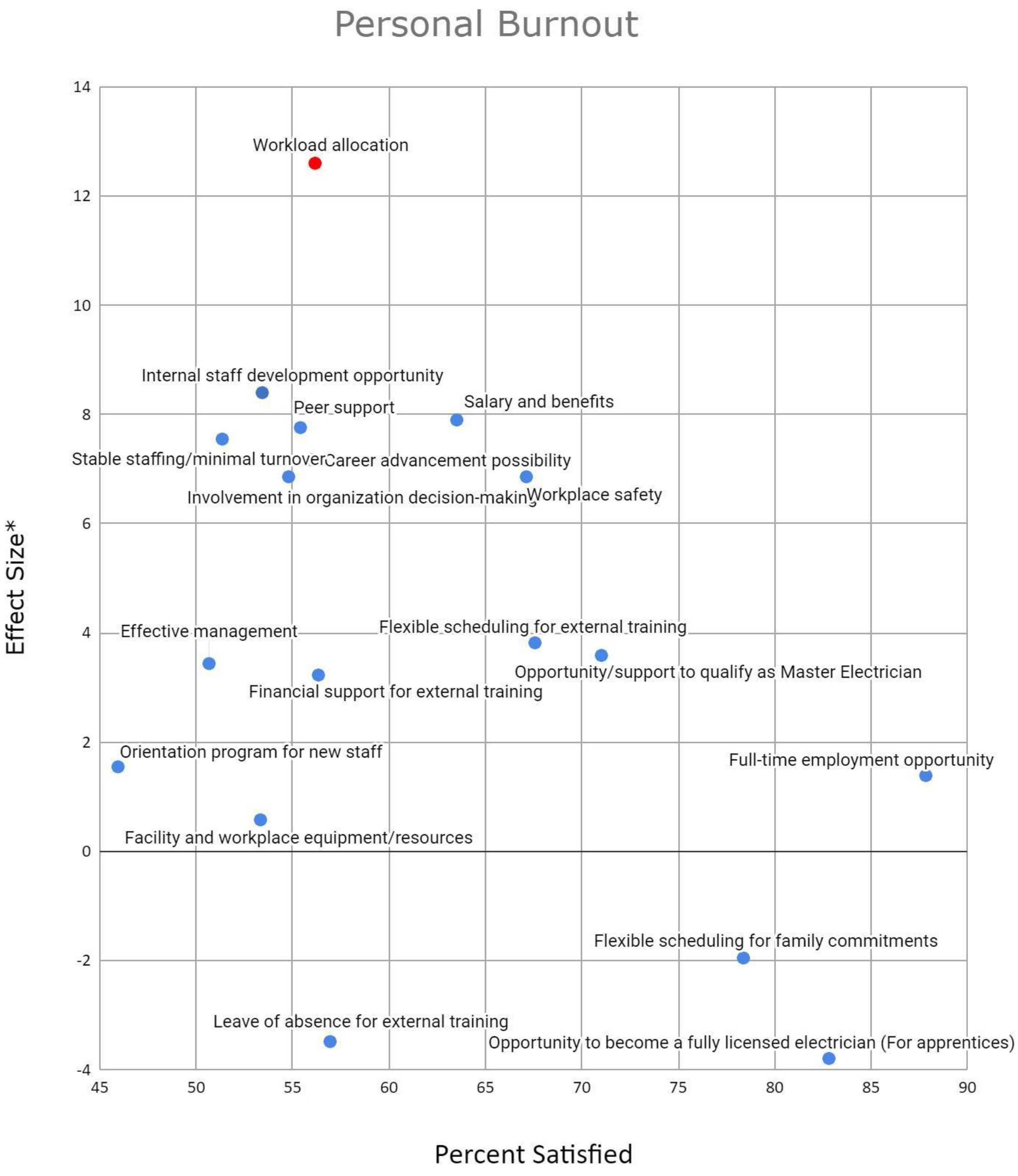

Figure S1. Impact of availability of work-related factors on personal burnout. * Effect size for a given work-related factor is calculated as the difference in average score of those dissatisfied with the factor and those satisfied with the factor. Red dots indicate the relationship between satisfaction with the factor and their average burnout score is significant to an alpha level of 0.05. Blue dots indicate the relationship is not significant to an alpha level of 0.05.

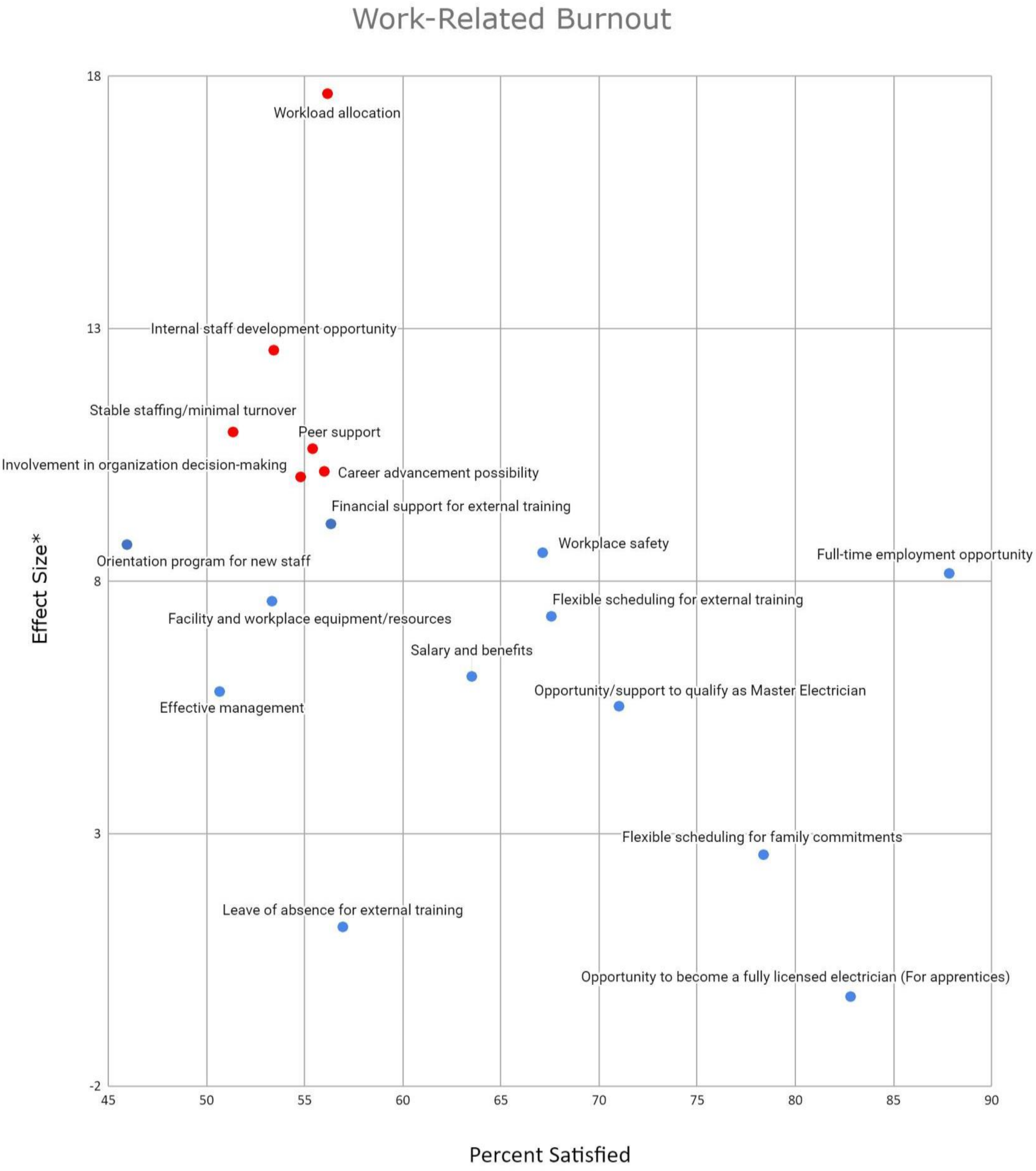

Figure S2. Impact of availability of work-related factors on work-related burnout. * Effect size for a given work- related factor is calculated as the difference in average score of those dissatisfied with the factor and those satisfied with the factor. Red dots indicate the relationship between satisfaction with the factor and their average burnout score is significant to an alpha level of 0.05. Blue dots indicate the relationship is not significant to an alpha level of 0.05.

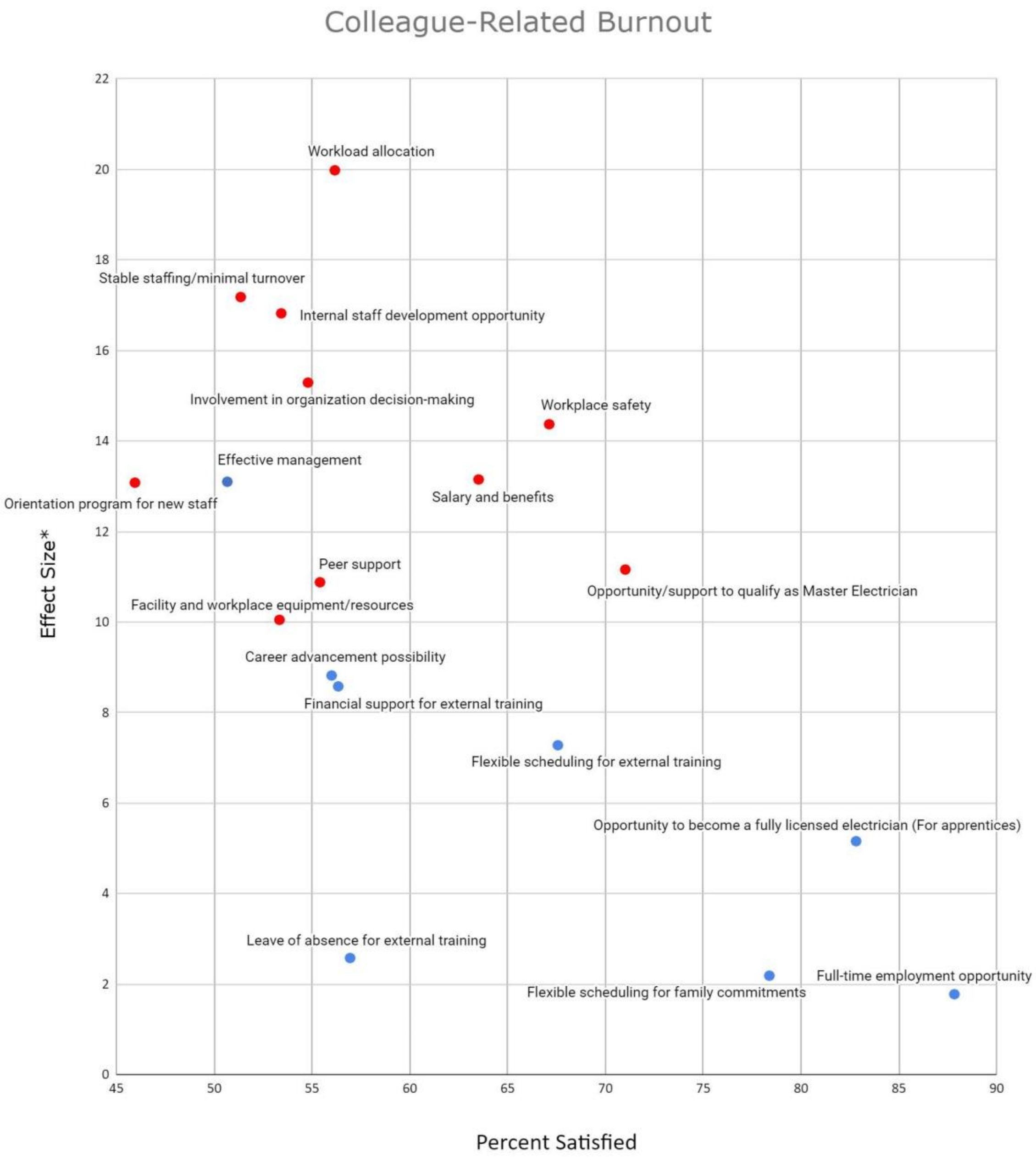

Figure S3. Impact of availability of work-related factors on colleague-related burnout. * Effect size for a given work- related factor is calculated as the difference in average score of those dissatisfied with the factor and those satisfied with the factor. Red dots indicate the relationship between satisfaction with the factor and their average burnout score is significant to an alpha level of 0.05. Blue dots indicate the relationship is not significant to an alpha level of 0.05.

Table S1. Association between the availability/satisfaction of work-related factors and the experience of burnout (n = 82).

Factors	Satisfied with availability of work-related factors (%)	Personal burnout				Work-related burnout				Colleague-related burnout				Total burnout			
		Mean non-satisfied	Mean satisfied	Mean difference*	p value**	Mean non-satisfied	Mean satisfied	Mean difference*	p value**	Mean non-satisfied	Mean satisfied	Mean difference*	p value**	Mean non-satisfied	Mean satisfied	Mean difference*	p value**
Career advancement possibility	56.0	42.02	34.85	7.17	0.16	43.70	33.52	10.18	0.04	42.94	34.12	8.82	0.08	43.74	33.49	10.25	0.04
Effective management	50.7	39.74	36.30	3.44	0.49	40.95	35.13	5.82	0.25	44.64	31.54	13.10	0.09	42.51	33.61	8.90	0.08
Facility and workplace equipment/resources	53.3	38.31	37.73	0.58	0.91	42.06	34.45	7.61	0.13	43.36	33.31	10.05	0.05	41.40	35.03	6.37	0.21
Financial support for external training	56.3	37.82	34.59	3.23	0.51	41.15	32.01	9.14	0.06	40.89	32.31	8.58	0.08	41.00	32.13	8.87	0.07
Flexible scheduling for external training	67.6	40.08	36.26	3.82	0.47	42.44	35.13	7.31	0.17	42.42	35.14	7.28	0.17	42.42	35.14	7.28	0.17
Flexible scheduling for family commitments	78.4	35.97	37.92	-1.95	0.75	39.53	36.94	2.59	0.67	39.22	37.03	2.19	0.72	37.53	37.49	0.04	1.00
Full-time employment opportunity	87.8	38.72	37.33	1.39	0.86	44.67	36.51	8.16	0.28	39.06	37.28	1.78	0.82	41.28	36.98	4.30	0.57
Internal staff development opportunity	53.4	41.49	33.09	8.40	0.09	43.72	31.14	12.58	0.01	45.99	29.17	16.82	<0.001	44.69	30.29	14.40	0.00
Involvement in organization decision-making	54.8	40.76	33.90	6.86	0.17	42.52	32.45	10.07	0.04	45.38	30.09	15.29	0.00	43.70	31.48	12.22	0.01
Leave of absence for external training	56.9	34.52	38.00	-3.48	0.48	37.16	36.00	1.16	0.82	37.97	35.39	2.58	0.60	36.89	36.21	0.68	0.89
Orientation program for new staff	45.9	38.21	36.66	1.55	0.76	41.51	32.78	8.73	0.08	43.51	30.43	13.08	0.01	41.51	32.78	8.73	0.08
Peer support	55.4	41.80	34.04	7.76	0.12	43.39	32.76	10.63	0.03	43.53	32.65	10.88	0.03	43.52	32.66	10.86	0.03
Salary and benefits	63.5	42.52	34.62	7.90	0.13	41.39	35.27	6.12	0.24	45.85	32.70	13.15	0.01	43.78	33.89	9.89	0.06
Stable staffing/minimal turnover	51.4	41.38	33.83	7.55	0.13	43.13	32.17	10.96	0.03	46.32	29.14	17.18	<0.001	44.50	30.87	13.63	0.01
Workload allocation	56.2	44.08	31.48	12.60	0.01	46.92	29.26	17.66	<0.001	48.22	28.24	19.98	<0.001	47.70	28.65	19.05	<0.001
Workplace safety	67.1	41.60	34.74	6.86	0.19	42.75	34.18	8.57	0.10	46.65	32.28	14.37	0.01	44.69	33.23	11.46	0.03
Opportunity to become a fully license electrician (For apprentices)	82.8	29.36	33.15	-3.79	0.54	32.32	32.54	-0.22	0.97	36.77	31.61	5.16	0.40	32.95	32.41	0.54	0.93
Opportunity/support to qualify as Master Electrician	71.0	37.55	33.96	3.59	0.50	38.93	33.40	5.53	0.30	42.93	31.77	11.16	0.04	40.50	32.76	7.74	0.15

Note: *: Mean difference (also referred to as "Effect size") is calculated as the mean burnout score by those not satisfied with the availability of a work-related factor subtract the mean burnout score of those satisfied with the work-related factor. Higher mean difference (positive) indicates that the dissatisfaction of a work-related factor is associated with a higher burnout score. ** The p-value was obtained from the Mann-Whitney U test.

Table S2. Comparison of burnout scores over a two years period.

**Type of burnout**	**Personal burnout**		**Work-related burnout**		**Colleague-related burnout**	
	**Mean [*SD*]**	**Burnout rate (%)***	**Mean [*SD*]**	**Burnout rate (%)***	**Mean [*SD*]**	**Burnout rate (%)***
**After year 1 (*n* = 40)**	33.48 [16.60]	17.5	25.63 [13.21]	5.0	17.56 [16.32]	5.0
**After year 2 (*n* = 82)**	40.51 [19.95]	33.3	33.02 [19.04]	19.8	23.29 [19.40]	13.6

Note: *Burnout rate indicates the percentage of respondents who have experienced either a moderate, a high, or a severe level of burnout.

AIMS Press

© 2023 the Author(s), licensee AIMS Press. This is an open access article distributed under the terms of the Creative Commons Attribution License (http://creativecommons.org/licenses/by/4.0)
